# Comment on: “Search and Selection of Probiotics that Improve Mucositis Symptoms in Oncologic Patients: A Systematic Review. *Nutrients* 2019, *11*, 2322”

**DOI:** 10.3390/nu12020399

**Published:** 2020-02-03

**Authors:** Claudio de Simone

**Affiliations:** 1660 Chateau d’Oex, Switzerland; claudio.desimone@bluewin.ch

I have read the systematic review by Picò et al. published in *Nutrients* 2019, *11*(10), 2322; https://doi.org/10.3390/nu11102322 and I noticed that the information reported in their article regarding the VSL#3 is not correct. The original formulation (the De Simone Formulation), the subject of over 70 clinical trials, has no longer been available as VSL#3^®^ since early 2016. I was surprised that the authors (whom I contacted privately), did not want to take any initiative to correct the wrong information. According to Dr Picò et al. “our work has been limited to discussing the methodological quality and results of 15 clinical trials and to comment on several preclinical studies. We can’t change anything about those articles (Friederich et al. 2011, Lacouture et al. 2016 and Bowen J.M. et al. 2007) because we don’t have authorization from the authors.” This is the position of the authors, despite the solid legal and scientific arguments showing that the brand VSL#3^®^, commercialized by Ferring in Europe and Alfasigma in the USA, refers to a product different from the original formulation utilized in the studies mentioned by Picò et al. in the review. To be clearer, the VSL#3^®^ presently marketed has never been studied in mucositis, gastrointestinal or liver diseases, or any other diseased state.

While the information reported in papers from Friederich, Lacouture and Bowen was appropriate for the formulation available at that time as VSL#3^®^, presently this is not true, considering the recent legal and scientific evidence [1,2,3,4,5]. For example, the Maryland District Court verdict on 20 June, 2019 issued a permanent injunction, prohibiting (1) stating or suggesting in VSL#3 promotional materials directed at or readily accessible to United States consumers that the present version of VSL#3 produced in ltaly (“Italian VSL#3”) continues to contain the same formulation found in the versions of VSL#3 produced before 31 January, 2016 (“the De Simone Formulation”), including but not limited to making statements that VSL#3 contains the “original proprietary blend” or the “same mix in the same proportions” as earlier version of VSL#3; and (2) citing to or referring to any clinical studies performed on the De Simone Formulation or earlier versions of VSL#3 as relevant or applicable to Italian VSL#3.

An issue in determining the utility and legitimacy of a review paper is whether it provides updated information for the benefit of the science or whether it maintains information which has been proven to be no longer accurate and is presently misleading. Should the authors remain entrenched in a crystallized information or assume the responsibility to adapt it according to new evidence?
